# Taurine Alleviates Pancreatic β‐Cell Senescence by Inhibition of p53 Pathway

**DOI:** 10.1111/1753-0407.70100

**Published:** 2025-06-03

**Authors:** Baomin Wang, Ziyan Wang, Yumei Yang, Melody Yuen Man Ho, Runyue Yang, Huizi Yang, Siyi Liu, Huige Lin, Kenneth King Yip Cheng, Xiaomu Li

**Affiliations:** ^1^ Department of Endocrinology and Metabolism Zhongshan Hospital Fudan University Shanghai China; ^2^ Fudan Institute for Metabolic Diseases, Fudan University Shanghai China; ^3^ Department of Health Technology and Informatics The Hong Kong Polytechnic University Hong Kong China; ^4^ The Hong Kong Polytechnic University Shenzhen Research Institute Hong Kong China

**Keywords:** cellular senescence, p53, pancreatic β‐cells, taurine

## Abstract

**Background:**

Pancreatic β‐cells function deteriorates during aging, leading to increased risk of type 2 diabetes. We and others previously demonstrated that p53 activation triggers β‐cell senescence and dysfunction in aging, but how its activity is controlled remains incompletely understood. Metabolites are not only by‐products of metabolic pathways but also function as messengers to regulate various biological pathways. Taurine, a non‐proteinogenic amino acid derived from cysteine, has demonstrated anti‐aging effects in multiple cell types and tissues. Nevertheless, its role in β‐cell senescence remains unclear.

**Methods:**

Untargeted metabolomic analysis was used to determine differential metabolites in pancreatic islets of mice during aging. In vitro, β‐cell lines MIN6 and INS‐1E were treated with taurine and its transporter inhibitor, followed by measurement of senescence‐related markers. Multiple experimental techniques, such as LC–MS/MS, co‐immunoprecipitation, DARTS analysis, and LiP‐MS, were used to study the mechanistic actions of taurine.

**Results:**

Untargeted metabolomic analysis showed that taurine and taurocholic acid were significantly upregulated in aged islets. Pretreatment with taurine inhibited naturally aging, chemically induced senescent and inflammatory program, oxidative stress, and defective insulin secretion in pancreatic β‐cells. SLC6A6 transporter was required to mediate exogenous taurine uptake, and inhibition of SLC6A6 abolished the anti‐senescent effects of taurine. Taurine bound with CKDN2AIP and inhibited its interaction with p53, thereby promoting p53 degradation and suppressing the p53‐dependent senescent program.

**Conclusion:**

Our findings suggest that increasing β‐cell taurine uptake might be a feasible approach to preserve β‐cell function by targeting the p53‐dependent senescent response.

## Graphical abstract

Taurine in β cell senescence.

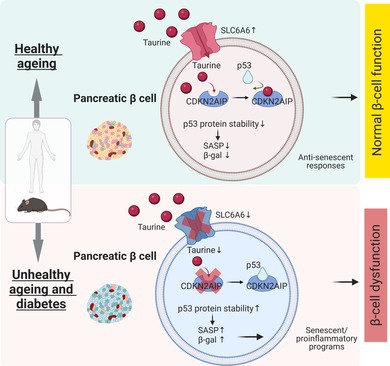

**Summary**

- Taurine and its transporter SLC6A6 are upregulated in pancreatic β‐cells in aging, but are diminished under diabetic conditions.
- Treatment with taurine largely abolishes DNA damage and pro‐inflammatory cytokine‐induced p53‐dependent senescent responses in rodent pancreatic β‐cells.
- Inhibition of taurine uptake by inactivating SLC6A6 abolishes the anti‐senescent actions of taurine. Taurine binds to the positive regulator of p53, CDKN2AIP, thereby inhibiting the p53‐CDKN2AIP interaction and subsequent p53 expression.

**Abbreviations**
BCAA: branched chain amino acids
DARTS: drug affinity responsive target stability
IL‐1β: interleukin 1β
LC–MS/MS: liquid chromatography–tandem mass spectrometry
LiP‐MS: limited proteolysis mass spectrometry
LXRα: liver X receptor α
OPLS‐DA: orthogonal projection to latent structures discriminant analysis
PCA: principal component analysis
Pdk1: 3‐phosphoinositide‐dependent protein kinase‐1
ROS: reactive oxygen species
SASP: senescence‐associated secretory phenotype
T2DM: type 2 diabetes mellitus
TNF‐α: tumor necrosis factor‐alpha

## 1 Introduction

The prevalence of type 2 diabetes mellitus (T2DM) is rising globally, driven by a complex interplay of genetic, nutritional, environmental, and lifestyle factors [1]. According to the International Diabetes Federation, the number of individuals with diabetes has surpassed 400 million, and this figure is expected to continue to increase in the coming decades [2, 3, 4, 5]. Pancreatic β‐cells maintain systemic glucose homeostasis via production and secretion of insulin in response to nutrient stimulation. However, β‐cell function deteriorates with aging, and this contributes to a high incidence of T2DM in the aging population [6, 7, 8, 9].

Senescent cells gradually accumulate in almost all tissues during aging, which disrupt normal tissue functions. Pancreatic β‐cells also undergo senescence, as featured by the senescence‐associated secretory phenotype (SASP), cell cycle arrest, inflammation, DNA damage, and oxidative stress. The SASP includes the production of pro‐inflammatory cytokines, such as tumor necrosis factor‐alpha (TNF‐α) and interleukin 1β (IL‐1β), and stress factors such as GDF15, which exacerbate β‐cell dysfunction and promote inflammation within the pancreatic microenvironment [10]. Several risk factors, including hyperglycemia, pro‐inflammatory cytokines, and DNA damage agents, are known to accelerate β‐cell senescence and trigger T2DM. p53 is a common activator of the senescent response, which initiates p21‐dependent cell cycle arrest and pro‐inflammatory cytokine expression. We recently showed that the reduction of the mitochondrial enzyme pyruvate carboxylase leads to diminished MDM2 expression and subsequent p53 overactivation and senescence in human and rodent islets during aging [11, 12, 13]. Given that β‐cell metabolism dynamically alters in response to aging and nutritional status, whether other metabolic pathways are involved in the senescent response remains poorly understood.

Metabolomic analysis has identified not only small metabolites as biomarkers but also etiological factors for T2DM [14, 15]. For example, circulating branched chain amino acids (BCAA) are known to be elevated in T2DM and cause insulin resistance under the obese condition [16]. While transcriptomic and proteomic approaches have been extensively utilized to elucidate β‐cell functionality in prior investigations, few of them employed metabolomics to assess β‐cells, in particular under aging conditions [17, 18, 19, 20, 21, 22]. In this study, we employed an in‐house high‐throughput metabolomics approach to profile hundreds of metabolites in pancreatic islets isolated from young and aged C57BL6/J mice. We demonstrated that metabolite profiles are completely distinct between young and aged islets. Taurine, one of the highly upregulated metabolites, prevents β‐cell senescence and dysfunction. Importantly, taurine metabolism is also altered in human islets under aging and diabetic conditions.

## 2 Materials and Methods

### 2.1 Human Pancreas Studies

Human pancreatic tissues were obtained from non‐diabetic and diabetic donors of various ages by the Department of Liver Surgery and Transplantation at Zhongshan Hospital affiliated with Fudan University between 2022 and 2024. The study was approved by the Ethics Committee of Zhongshan Hospital (Ethics approval number: B2021‐853R). Written informed consent was procured from all participants, and all procedures adhered to the Declaration of Helsinki. Clinical and metabolic characteristics of the donors are detailed in Tables S1, S2. Diabetes was diagnosed according to the American Diabetes Association (ADA) standard clinical criteria of HbA1C ≥ 6.5%. The pancreatic tissues were stored in chilled preservation solution before further processing. The pancreas was dissected and rinsed with phosphate‐buffered saline (PBS). Small pieces of tissue were then either snap‐frozen in liquid nitrogen for molecular analysis or fixed in formalin for histological studies. Frozen tissues were stored at −80°C until use.

The pancreas was perfused via the pancreatic duct with collagenase P (0.4 mg/mL, Roche, Cat#11213865001) prepared in Hank's Balanced Salt Solution (HBSS). Following perfusion, the pancreas was incubated at 37°C for 15–20 min with gentle agitation to facilitate digestion. Subsequently, the isolated islets were subjected to density gradient centrifugation using Histopaque 1119 (Sigma‐Aldrich, Cat# 1119–1) and 1077 (Sigma‐Aldrich, Cat#1077‐1) at 4°C and 339 × *g* for 30 min. Purified human islets collected between the second and third layers of the gradient were manually retrieved under a microscope. These isolated islets were cultured in RPMI 1640 medium (Gibco, Cat#11879020) supplemented with 5.5 mM glucose, 10% fetal bovine serum (FBS), 50 μM β‐mercaptoethanol (MCE, Cat#HY‐Y0326), and antibiotics (penicillin–streptomycin, 100 U/mL and 100 μg/mL, Gibco, Cat# 15140‐122).

### 2.2 Isolation of Primary Pancreatic Islets From Mice

Male C57BL/6 mice at different ages were used for islet isolation. The mice were fasted for 4–6 h before sacrifice. The pancreas was perfused with collagenase P (0.4 mg/mL) prepared in HBSS via the pancreatic duct, followed by incubation at 37°C for 15–20 min with gentle agitation. After digestion, the tissue was washed with HBSS supplemented with 0.1% bovine serum albumin (BSA) and centrifuged at 300 × g to pellet the islets. The supernatant was discarded, and the pellet was resuspended in HBSS with 0.1% BSA. The islets were purified using a density gradient (Histopaque 1077 and 1119) by centrifugation at 339 × *g* for 30 min. The purified islets were collected and cultured in RPMI 1640 medium supplemented with 5.5 mM glucose, 10% FBS, 50 μM β‐mercaptoethanol, and 1% penicillin–streptomycin overnight at 37°C in a 5% CO_2_ incubator before untargeted and targeted metabolomics. All animal experiments were approved by the Department of Health, HKSAR Government (Ordinance Cap. 340), and the Animal Subjects Ethics Sub‐Committee (ASESC) of the Hong Kong Polytechnic University (24–25/1301‐HTI‐R‐RFS).

### 2.3 Cell Culture

Mouse pancreatic β‐cell line MIN6 (AddexBio Technologies, Cat#C0018008) and rat insulinoma cell INS‐1E (AddexBio Technologies, Cat#C0018009) were cultured in DMEM (Gibco, Cat#12800082) or RPMI 1640 supplemented with 15% FBS, 1% penicillin–streptomycin, and 50 μM β‐mercaptoethanol. The cells were maintained at 37°C in a humidified atmosphere with 5% CO_2_. Cells were passaged every 2–3 days using 0.05% trypsin–EDTA (Gibco, Cat# T4049). For experiments, β‐cells or isolated islets were seeded in appropriate culture vessels (plates or dishes) and allowed to attach and grow for 16 h before treatment with various agents, including taurine (100 μM, Beyotime, Cat#ST1686), hypotaurine (100 μM, MCE, Cat#HY‐100803), SLC6A6 inhibitor taurocyamine (100 μM, MCE, Cat#HY‐113329), doxorubicin (200 nM, MCE, Cat#HY‐15142A), or TNF‐α (20 ng/mL, Abcam, Cat#ab259410) for 24–48 h as indicated in each figure legend. For p53 protein degradation analysis, MIN6 cells were treated with protein synthesis inhibitor cycloheximide (CHX) (100 μg/mL, MCE, Cat#HY‐12320) for different time points as indicated in the figures. Protein samples were collected for immunoblotting analysis.

### 2.4 Taurine Measurement by LC–MS/MS

Intracellular taurine levels were quantified using liquid chromatography–tandem mass spectrometry (LC–MS/MS). The cells were washed twice with ice‐cold PBS and then extracted with 0.5 mL of ice‐cold 80% methanol containing an internal standard (valine‐d8, Cambridge Isotope Laboratories, Cat#DLM‐311‐PK). The lysates were vortexed, sonicated for 1 min, and centrifuged at 15000 × g for 20 min at 4°C. The supernatants were transferred to new tubes and evaporated to dryness under a vacuum concentrator. The residues were reconstituted in 100 μL of cold 80% methanol. LC–MS/MS analysis was performed using an Agilent 6460 Liquid Chromatography–Electrospray Ionization Triple Quadrupole Mass Spectrometer (University Life Science, The Hong Kong Polytechnic University) equipped with an electrospray ionization (ESI) source. The separation was achieved on the Acquity UPLC BEH Amide Column (1.7 μm, 2.1 × 100 mm, Waters, Cat# 186004801) using a binary mobile phase system consisting of 5 mM ammonium formate (Sigma‐Aldrich, Cat#09735) and 0.1% formic acid (Sigma‐Aldrich, Cat#695076) in mobile phase A (water: acetonitrile, 90:10, vol/vol) and mobile phase B (water: acetonitrile, 10:90, vol/vol). The flow rate was set at 0.3 mL/min, and the injection volume was 2 μL. The mass spectrometer was operated with the voltage set to 3.5 kV and source temperature held at 300°C. The target analytes were monitored by multiple reaction monitoring (MRM) in positive ESI mode with the specific precursor ion‐to‐product ion transitions determined for each metabolite. The fragmentor voltage (80–90 V) and collision energy (8–15 psi) were also optimized for each metabolite to facilitate their detection and quantification. The acquired mass spectra data were analyzed using Agilent MassHunter Quantitative Analysis Software. Enrichment of metabolites was corrected by internal standards and normalized to the protein concentration of the samples.

### 2.5 Plasmid Constructs

Human GFP‐CDKN2AIP wildtype plasmid and the CDKN2AIP‐triple mutant: P484A/L485A/K486A‐CDKN2AIP were generated by Vigene Bioscience. All plasmids and mutations were validated by DNA sequencing.

### 2.6 Plasmid and siRNA Transfection

For siRNA transfection, approximately 1 × 10^5^ MIN6 cells were seeded in 6‐well plates and cultured overnight to reach 50%–70% confluence. The following day, the cells were transfected with siRNA targeting *Slc6a6* or a non‐targeting control siRNA (so‐called scramble hereafter) using Lipofectamine RNAiMAX reagent (Invitrogen, Cat#13778150) according to the manufacturer's instructions. The sequences of siRNA are listed in Table S3. For plasmid transfection, ~1 × 10^5^ HEK293/INS‐1E cells were seeded in 6‐well plates and cultured overnight to reach 50%–70% confluence. The following day, the cells were transfected with plasmids encoding GFP‐tagged CDKN2AIP (WT) and CDKN2AIP‐triple mutant (MT) using Lipofectamine 3000 reagent (Invitrogen, Cat# L3000015) according to the manufacturer's instructions.

### 2.7 In Vitro Assays for Mitochondrial Function

Mitochondrial membrane potential in MIN6 cells was determined using tetramethylrhodamine ethyl ester (TMRE) membrane potential assay (Cayman Chemical, Cat#701310) with 50 nM TMRE. Fluorescence was measured using BMG LABTECH CLARIO star with excitation and emission wavelengths at 530 and 580 nm, respectively.

### 2.8 Measurement of Lipid Peroxidation via Malondialdehyde (MDA) Assay

Lipid peroxidation was assessed by measuring malondialdehyde (MDA) levels using the thiobarbituric acid reactive substances (TBARS) method. MIN6 cell lysates were mixed with SDS, acetic acid, and thiobarbituric acid, heated at 95°C for 60 min, and centrifuged. Absorbance at 532 nm was measured spectrophotometrically. MDA concentration was calculated against a 1,1,3,3‐tetramethoxypropane (TMP) standard curve and normalized to total protein content.

### 2.9 Co‐Immunoprecipitation

Cells were lysed in an immunoprecipitation buffer (50 mM Tris–HCl, pH 7.4, 150 mM NaCl, 1% NP‐40, 0.5% sodium deoxycholate, 0.1% SDS, protease inhibitor cocktail) on ice for 30 min and then centrifuged at 14000 × *g* for 15 min at 4°C. The supernatant was collected, and the protein concentration was determined using a BCA protein assay kit (Thermo Fisher Scientific, Cat#23225). For immunoprecipitation, an appropriate amount of cell lysate (500–1000 μg protein) was incubated with an anti‐CDKN2AIP antibody (Proteintech, Cat#16615‐1‐AP) or control IgG overnight at 4°C with gentle rotation. Protein A/G agarose beads (Millipore, Cat# IP10) prewashed with immunoprecipitation buffer were then added to the mixture and incubated for 2–4 h at 4°C. The beads were washed three times with immunoprecipitation buffer and then eluted with 2X SDS sample loading buffer by boiling for 5 min. The eluted proteins were resolved by SDS‐PAGE and analyzed by immunoblotting using specific antibodies.

### 2.10 Drug Affinity Responsive Target Stability (DARTS) Analysis

DARTS was performed as previously described with some modifications [23]. Briefly, MIN6 or INS‐1E cells were washed with PBS and lysed in lysis buffer (20 mM Tris–HCl, 150 mM NaCl, 1% Triton X‐100, pH 7.5, protease inhibitor cocktail). The lysates were centrifuged at 15000 × *g* for 15 min at 4°C, and the supernatants were collected. Protein concentration was determined using a BCA protein assay kit. Equal amounts of protein (50–100 μg) were incubated with increasing concentrations of taurine (0–200 μM) at a final volume of 50 μL for 1 h on ice. After incubation, 1 μL of 100 μg/mL proteinase K (Sigma‐Aldrich, Cat# SAE0151) was added to each sample, and the mixtures were incubated for an additional 5 min at room temperature. The digestion was stopped by heating at 95°C for 10 min with SDS loading buffer.

### 2.11 Limited Proteolysis Small Molecule Mapping (LiP‐MS) Approach

LiP‐MS was performed according to a previously reported method with some modifications [24]. INS‐1E cells were lysed in lysis buffer (100 mM HEPES, 150 mM KCl, 1 mM MgCl_2_, pH 7.5) and centrifuged at 20 000 × *g* for 5 min at 4°C. Endogenous small molecules were removed using size‐exclusion chromatography with Zeba Spin Desalting Columns (7 MWCO, Thermo Fisher Scientific, Cat#89890). Protein concentration was determined by BCA. Lysates containing 300 μg of protein were aliquoted into tubes and incubated with taurine (1 mM in 1 mM HEPES, pH 7.5) for 10 min at 25°C. Proteinase K was added at a ratio of 1:100 (proteinase K to protein) and incubated at 25°C for 5 min. Limited digestion was stopped by heating at 98°C for 3 min. To enhance protein solubilization and facilitate downstream analysis, sodium deoxycholate was added to a final concentration of 5% and the mixture was heated at 98°C for an additional 3 min.

Following limited proteolysis, samples were processed using the EasyPep 96 MS Sample Prep Kit (Thermo Fisher Scientific, Cat#A45733). Reduction and alkylation solutions were added sequentially, followed by heating at 95°C for 10 min. The samples were cooled, and a reconstituted Trypsin/Lys‐C protease mix was added before incubation at 37°C for 16 h. Digestion was stopped with the Digestion Stop Solution. The digested peptides were transferred and cleaned using the peptide clean‐up plate from the kit, followed by centrifugation. The eluted peptides were dried in a vacuum centrifuge and resuspended in 0.1% formic acid for mass spectrometry analysis.

Peptides were analyzed using a Thermo Scientific Orbitrap Fusion Lumos Mass Spectrometer equipped with a nano‐electrospray ion source and an Ultimate 3000 RSLCnano liquid chromatography system. Injection volume was set to 1 μL. The peptides were analyzed using a data‐dependent acquisition (DDA) strategy in positive ion mode, with a scan range spanning from 400 to 1500 m/z and a resolution of 60 000 under standard automatic gain control (AGC) conditions. Ions with multiple charge states between 2 and 6 were utilized to trigger MS–MS scans, while the dynamic exclusion duration was set to 40 s. Selected precursors were fragmented via high‐energy collision dissociation (HCD), with the normalized collision energy adjusted to 30%. The MS/MS spectra were acquired using an Orbitrap mass analyzer at a mass resolution of 7500 under standard AGC settings. The mass spectrometry proteomics data were deposited into the ProteomeXchange Consortium through the PRIDE partner repository. Among the identified targets, those with abundances correlating with increased taurine concentrations were singled out for further investigation.

### 2.12 Histological Studies

Human pancreatic tissues were fixed in 4% paraformaldehyde overnight at 4°C, dehydrated using a graded ethanol series, and embedded in paraffin. Sections (5 μm thick) were cut using a microtome and mounted on glass slides. For immunohistochemistry, the sections were deparaffinized in xylene and rehydrated through a graded ethanol series. Antigen retrieval was performed by heating the sections in citrate buffer (pH 6.0) in a microwave oven for 10 min. After cooling, the sections were blocked with 5% BSA in PBST for 1 h at room temperature and then incubated with primary antibody (anti‐SLC6A6, Proteintech, Cat# 26725‐1‐AP) overnight at 4°C. The sections were washed with PBST and incubated with horseradish peroxidase (HRP)‐conjugated anti‐rabbit (CST, Cat#7074S) for 1 h at room temperature. The immunoreactivity was visualized using a 3,3′‐diaminobenzidine (DAB) substrate kit (Abcam, Cat#ab64238), and the sections were counterstained with hematoxylin. Images were captured using a bright‐field microscope Nikon Y‐THPL Microscope and analyzed using ImageJ software. For senescence‐associated‐β‐gal staining, SA‐beta‐gal activity of isolated islets was detected using the senescence β‐galactosidase staining kit (CST, Cat# 9860) according to the manufacturer's instructions.

### 2.13 Flow Cytometry Assay of β‐Gal

MIN6 cells were detached using trypsin and resuspended in 1X PBS, followed by incubation with 2% paraformaldehyde for 10 min at room temperature. The cells were then stained with a fluorescent green probe (1;500; Thermo, Cat#C10840) to detect senescence according to the manufacturer's protocol. The staining process was conducted in a 37°C incubator without carbon dioxide supply for 30 min, followed by washing with 1X PBS supplemented with 1% BSA. The cells were then resuspended in 1X PBS. To evaluate cell viability, the stained MIN6 cells were further incubated with propidium iodide (PI, 20 μg/mL) (Thermo, Cat#P1304MP) in the dark at room temperature for 15 min. The stained cells were then analyzed using a BD FACSCelesta flow cytometer, and the obtained data were processed and analyzed using FlowJo software.

### 2.14 Immunoblotting Analysis

Cells were homogenized in a RIPA lysis buffer formulated with 150 mM NaCl, 50 mM Tris HCl at pH 7.4, 2 mM EDTA, 0.1% SDS, and 1% NP‐40. Protease inhibitors (MCE, Cat#HY‐K0010) and phosphatase inhibitors (Selleck Chemicals, Cat#B15002) were added to the buffer. Equal amounts of protein were then separated by SDS‐PAGE and transferred onto a PVDF membrane (Millipore, Cat# HATF00010). Subsequently, the membrane was blocked with 5% non‐fat milk and incubated with primary antibodies against SLC6A6, CDKN2AIP, MDM2 (Millipore, Cat#04‐1530), p53 (Proteintech, Cat#10442‐1‐AP), p21 (Abcam, Cat# ab188224), β‐actin (Santa Cruz, Cat#sc‐47 778), HSP90 (Proteintech, Cat#13171‐1‐AP) overnight at 4°C.

After that, the membranes were washed four times with TBS‐T. This was followed by one‐hour incubation with the corresponding secondary antibodies conjugated with horseradish peroxidase (Cell Signaling Technology, Cat#7074 and 7076) at room temperature. For chemiluminescent detection, the membrane was reacted with Clarity Western ECL substrate (Bio‐Rad, Cat#170‐5061) for 4 min as per the manufacturer's instructions. Finally, the intensity of the protein bands on the film was quantified using ImageJ software.

### 2.15 Real‐Time Quantitative PCR Analysis

Total RNA was extracted using TRIzol (Takara, Cat#15596026) following the manufacturer's guidelines. 1 μg of RNA and random universal hexamer primers were used for reverse transcription using a GoScript Reverse Transcription kit (Promega, Cat#A2801). The synthesized cDNA (1:40 dilution) was mixed with gene‐specific primers and SYBR Green reaction buffer (Qiagen, Cat#208056) for quantitative PCR analysis, which was monitored in the ViiA7 Real‐time PCR System (Applied Biosystems). To obtain accurate and comparable results, the expression levels of target genes were calibrated against at least two housekeeping genes, such as ACTIN, 36B4, and 18S, as denoted in the figure legends. Details of the primer sequences are listed in Table S4.

### 2.16 Untargeted Metabolomics of Young and Aged Mouse Islets

Islets were isolated from 2‐month‐old (26 mice) and 18‐month‐old (24 mice) C57BL/6 mice, yielding a total of 5400 islets for the young group and 4700 islets for the aged group. These islets were divided based on cell numbers, resulting in 5 samples in the young group and 4 samples in the aged group, with each sample containing ~5.5 × 10^5^ cells. For the untargeted metabolomic analysis, 4 mL of 80% HPLC‐grade methanol (pre‐cooled to −80°C) was added to the cell pellet. Following vortexing for 1 min and incubation at −80°C for 30 min, the samples were centrifuged at 4000 × *g* for 10 min at 4°C. The supernatant was then collected and dried, and stored at −80°C until mass spectrometry (MS) analysis. The dried samples were resuspended in 80 μL of water: acetonitrile (vol:vol, 50:50) and centrifuged at 14000 × *g* for 10 min at 4°C. The supernatant was transferred to autosampler vials for liquid chromatography‐mass spectrometry (LC–MS) analysis. A quality control (QC) sample was prepared by pooling equal volumes of each sample to evaluate the reproducibility of the LC–MS analysis. Following this, 2 μL of the samples was injected into a 7600 SCIEX ZenoTOF mass spectrometer.

MetaboAnalyst 6.0, accessible at “https://www.metaboanalyst.ca” was utilized for untargeted metabolomic data analysis. The data was log2 transformed and Pareto scaled to account for variance. Principal Component Analysis (PCA) and Orthogonal Projection to Latent Structures Discriminant Analysis (OPLS‐DA) were employed to discern differences between young and old groups. Metabolites with VIP scores > 1 were deemed significant in distinguishing the two groups. The differences in peak intensity of metabolites between the two groups were analyzed using *t*‐test. Metabolites were considered differentially expressed based on the criteria of |log2Fold Change (FC)| > 1, FDR < 0.05, and VIP score > 1. These differentially expressed metabolites were then subjected to KEGG enrichment analysis (http://www.genome.jp/kegg/) to further elucidate their biological significance.

### 2.17 Bioinformatic Analysis

The RNA sequencing data were derived from the “Human Islet Dataset” published in Cell Metabolism [25], with raw data available in the European Genome‐phenome Archive (EGA): EGAS00001007241. The samples used for this analysis were human islets from donors matched for sex and BMI, and subsequently divided into three groups based on age and HbA1c levels: Young non‐DM (age < 35 years, HbA1c < 5.9%, *n* = 13), Aged non‐DM (age ≥ 60 years, HbA1c < 5.9%, *n* = 14), and Aged DM (age ≥ 60 years, HbA1c ≥ 6.5%, *n* = 4). To discern differences in the expression levels of key genes related to taurine synthesis and transport among the three groups, one‐way ANOVA was employed.

### 2.18 Statistical Analysis

Data processing was carried out using GraphPad Prism 9.0. The results were expressed as mean ± SEM. To assess the statistical significance in two‐group comparisons, an unpaired Student's t‐test was employed for normally distributed data with equal variance, otherwise nonparametric Mann–Whitney *U*‐test was employed. In cases involving multiple group comparisons, a one‐way or two‐way ANOVA with Tukey post hoc test was utilized. The assumptions of equal variance and normality were verified by Levene's test and D'Agostino–Pearson omnibus normality test, respectively. A *p*‐value lower than 0.05 was regarded as statistically significant, and the specific statistical test used was detailed in each figure legend. The in vitro experiments were replicated independently two to three times, yielding consistent outcomes.

## 3 Results

### 3.1 Metabolome Profile of Mouse Pancreatic Islets in Natural Aging

**FIGURE 1:**
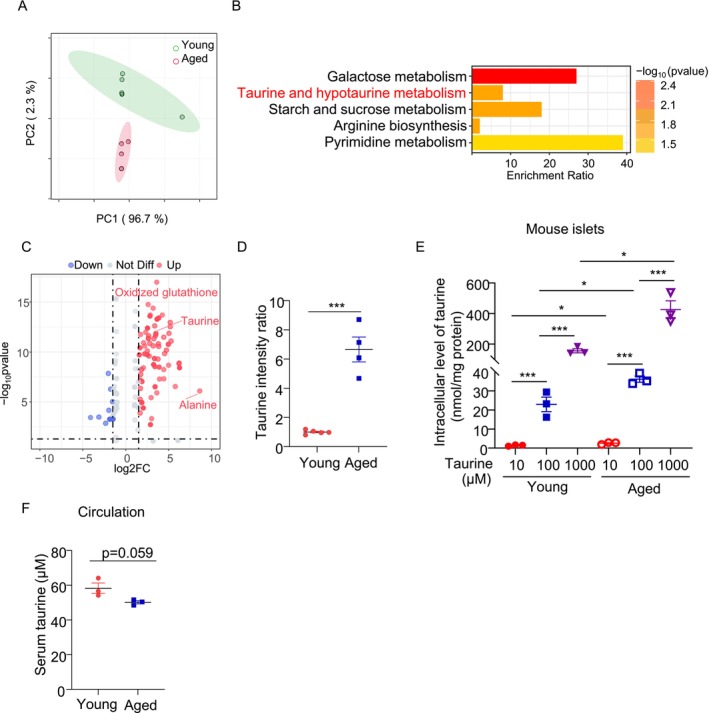
Taurine is upregulated in pancreatic β‐cells during natural aging. (A–D) Islets isolated from 26 C57BL6/J mice aged 2 months (“Young”) and 24 mice aged 18 months (“Aged”) were pooled and subjected to untargeted metabolomics analysis. (*n* = 4–5). (A) Principal component analysis (PCA) on the metabolomic profiles of two groups. (B) KEGG pathway analysis. (C) Volcano plot showed the differentially expressed metabolites in young and aged islet metabolomics data. (D) Taurine expression between two groups. (E) Islets isolated from young and aged mice were treated with taurine (10, 100, and 1000 μM) for 24 h in non‐FBS culture medium. The intracellular taurine concentration was then measured by LC–MS/MS. (*n* = 3). (F) Taurine level in serum from young and aged mice. (*n* = 3–4). All results are presented as mean ± SEM. Significance was determined using two‐tailed independent student's *t*‐test for panels D and F; One‐way ANOVA with Tukey correction for panel E.*p < 0.05, **p < 0.01, ***p < 0.001.

To investigate alterations in metabolite profiles of pancreatic β‐cells in aging, we utilized naturally aged mice (18 months old, equivalent to ~56–69‐years‐old in humans) and young control mice (2 months old, equivalent to ~20–30‐years‐old in humans) [26]. Pancreatic islets were isolated from both groups and subjected to untargeted metabolomics analysis, identifying a total of 541 metabolites. Principal component analysis (PCA) revealed distinct clustering of metabolite expression profiles between aged and young islets, indicating significant metabolic differences (Figure 1A). To identify the differentially expressed metabolites between the two groups, independent sample t‐tests were performed with a significance level of *p* < 0.05. To reduce the false discovery rate, we employed the Benjamini‐Hochberg procedure for correction. Metabolites with a log2FC > 1 were categorized as upregulated, while those with log2FC < −1 were considered downregulated. This analysis identified 205 significantly altered metabolites, including 149 upregulated and 56 downregulated in aged islets. For further analysis and visualization of the differential distribution of metabolites, we employed Orthogonal Projection to Latent Structures Discriminant Analysis (OPLS‐DA) to enhance the accuracy of group classification. The model was validated through cross‐validation to ensure its stability and reliability. Ultimately, we identified 152 differentially expressed metabolites (128 upregulated and 24 downregulated) with a significance level of *p* < 0.05 and the VIP value ≥ 1. These metabolites were subjected to KEGG pathway enrichment analysis, revealing their involvement in galactose metabolism, taurine and hypotaurine metabolism, starch and sucrose metabolism, arginine biosynthesis, and pyrimidine metabolism (Figure 1B).

Taurine (2‐aminoethanesulfonic acid), a non‐proteinogenic amino acid derived from cysteine, is widely distributed in animal tissues [27]. Taurine deficiency has been shown to accelerate the aging process, whereas supplementation of taurine may confer protective effects [28, 29, 30]. Although taurine has been shown to protect β‐cells against oxidative stress, inflammation, and apoptosis [31, 32, 33, 34], its role in β‐cell senescence is unknown. Our untargeted metabolomics analysis showed that taurine was significantly upregulated in the aging islets when compared to those from young counterparts (Figure 1C,D). To further validate this, we detected taurine in the islets from another batch of young and aged islets using targeted liquid chromatography–tandem mass spectrometry (LC–MS/MS) analysis. Taurine uptake was increased proportionally with extracellular taurine in both young and aged islets, and the increment was more pronounced in the aged islets (Figure 1E). Interestingly, serum taurine levels in aged mice exhibited a decreasing trend (Figure 1F), indicating a potential increase in taurine uptake or intracellular taurine biosynthesis in pancreatic islets under aging conditions.

### 3.2 Taurine Supplementation Alleviates β‐Cell Inflammation and Senescence in Age‐and Drug‐Induced Models Ex Vivo and In Vitro

**FIGURE 2:**
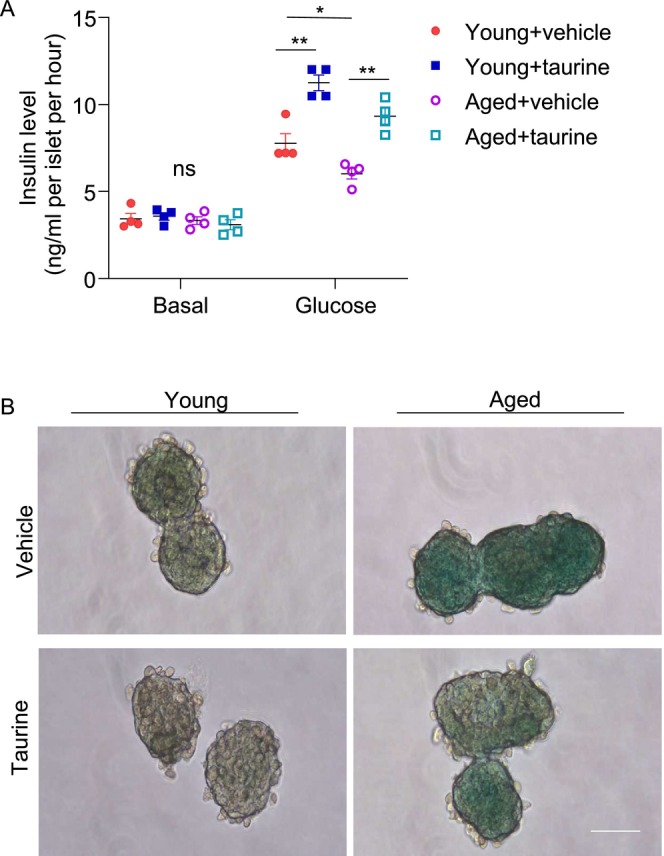
Beneficial effects of taurine on insulin secretion and senescence in young and aged islets. (A, B) Islets isolated from young (2‐month‐old) and aged (18‐month‐old) male mice were treated with vehicle or taurine (100 μM, 24 h). (A) Static GSIS analysis (*n* = 4). (B) Representative images of SA‐β‐gal staining (Scale bar: 100 μm). All results are presented as mean ± SEM. Significance was determined using two‐way ANOVA with Tukey correction. *p < 0.05, **p < 0.005, ***p < 0.001.

To explore whether taurine exerts beneficial effects on β‐cells under aging conditions, we employed different ex vivo and in vitro approaches. First, we isolated pancreatic islets from young and aged mice and then treated them with the physiological concentration of taurine (i.e., 100 μM) or vehicle control. Islets isolated from aged mice exhibited compromised glucose‐stimulated insulin secretion (Figure 2A). Concurrently, an elevated level of β‐galactosidase (β‐gal) positive signals, a hallmark of cellular senescence, was observed within the aged islets (Figure 2B). Taurine supplementation effectively mitigated β‐cell senescence and restored insulin secretory capacity in the aged islets (Figure 2A,B).

**FIGURE 3:**
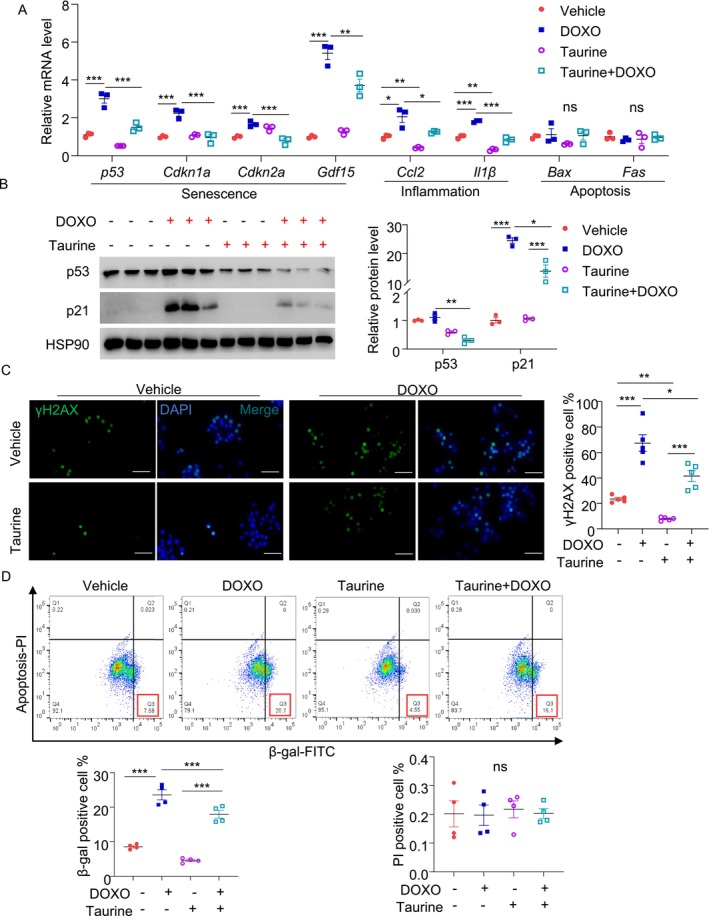
Taurine supplement alleviates doxorubicin‐induced β‐cell inflammation and senescence. MIN6 cells were pre‐treated with 100 μM taurine for 24 h, followed by 200 nM doxorubicin (DOXO) treatment for 24 h. Cells were cultured in FBS‐free medium to avoid possible contamination of taurine. (A) QPCR analysis of the genes related to inflammation, senescence, and apoptosis in each group of doxorubicin‐induced senescence model. (*n* = 3) Relative mRNA levels were normalized to β‐actin. (B) Immunoblotting analysis of p53 and p21 and densitometric quantification. (*n* = 3). (C) Immunofluorescence staining of DNA damage marker γ–H2AX in each group (scale bar: 100 μm). (*n* = 5). (D) FACS analysis of β‐gal+ PI‐(senescent) and PI+ (dead) MIN6 cells. All results are presented as mean ± SEM. Significance was determined using two‐way ANOVA with Tukey correction. *p < 0.05, **p < 0.01, ***p < 0.001.

Since DNA damage contributes to β‐cell senescence during aging [35], we mimicked this by treating MIN6 cells with the DNA damaging agent doxorubicin for 24 h. As expected, doxorubicin‐induced DNA damage (reflected by increased number of phospho‐γH2AX positive cells), accompanied by the induction of multiple senescent markers including mRNA and protein expression of p53 and its downstream Cdkn1a (p21; a cell cycle arrest protein), Cdkn2a (p16^Ink^) and the SASP, including *Gdf15*, *Ccl2*, and *Il‐1β*, as well as β‐gal positive signal. Pretreatment with taurine largely suppressed these senescent markers induced by doxorubicin (Figure 3A–D). Of note, the dosage of doxorubicin we used did not lead to changes in genes related to apoptosis (*Fas* and *Bax*) (Figure 3A) or cell death (Figure 3D), as assessed by QPCR and flow cytometry analyses, respectively. QPCR analysis also revealed that taurine supplementation significantly reduced the mRNA levels of *Il‐6* and *Mmp2*, while having minimal impact on *Mmp13* and *Mmp14* levels (Figure S1A). Additionally, taurine exerted the same anti‐senescent effects in rat INS‐1E β‐cells (Figure S1B).

**FIGURE 4:**
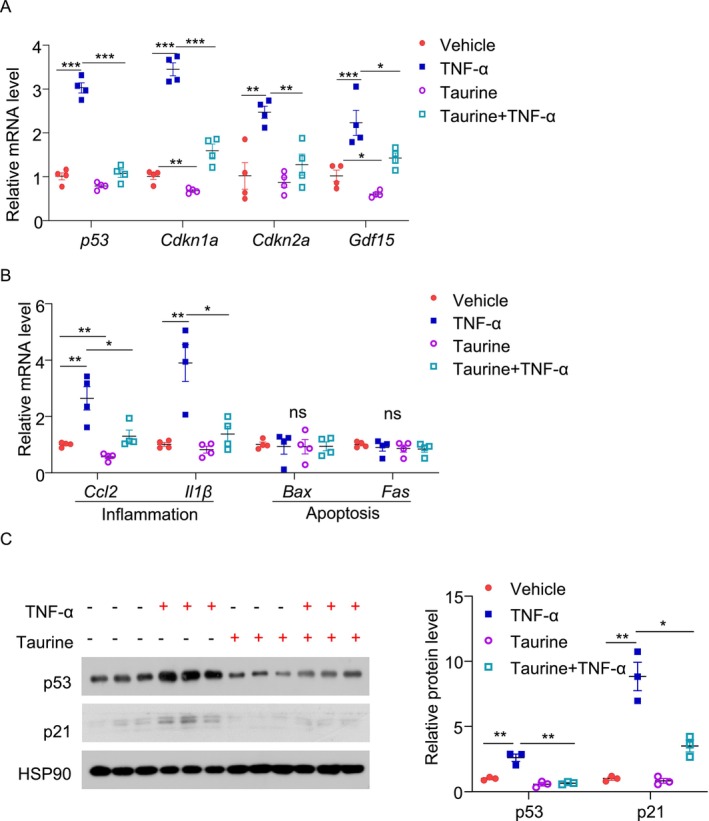
Taurine supplementation alleviates TNF‐α‐induced β‐cell inflammation and senescence. MIN6 cells were pre‐treated with 100 μM taurine for 24 h, followed by 20 ng/mL TNF‐α treatment for 24 h. Cells were cultured in FBS‐free medium to avoid possible contamination of taurine. (A) QPCR analysis of the genes related to senescence in each group of TNF‐α‐induced senescence model. (*n* = 4). Relative mRNA levels were normalized to β‐actin. (B) QPCR analysis of the genes related to inflammation and apoptosis in each group. (*n* = 4). Relative mRNA levels were normalized to β‐actin. (C) Immunoblotting analysis of p53 and p21 in each group and densitometric quantification. (*n* = 3). All results are presented as mean ± SEM. Significance was determined using two‐way ANOVA with Tukey correction. *p < 0.05, **p < 0.01, ***p < 0.001.

Apart from DNA damage, pro‐inflammatory cytokines such as TNF‐α have been shown to induce senescence in β‐cells during aging [36]. To generalize our findings, we examined whether taurine prevents TNF‐α‐induced senescence in β‐cells. Treatment with TNF‐α upregulated p53 expression and its related p21 expression, as well as several SASP including *Gdf15, Ccl2*, and *Il‐1β* in MIN6 cells but had minimal effect on apoptotic gene expression (Figure 4A–C). Pre‐treatment with taurine attenuated these pro‐senescence effects of TNF‐α (Figure 4A–C). Taken together, our findings suggest that taurine exerts a potent anti‐senescent effect in pancreatic β‐cells.

### 3.3 SLC6A6‐Mediated Taurine Uptake Is Enhanced in Senescent β‐Cells as an Anti‐Aging Adaptation

**FIGURE 5:**
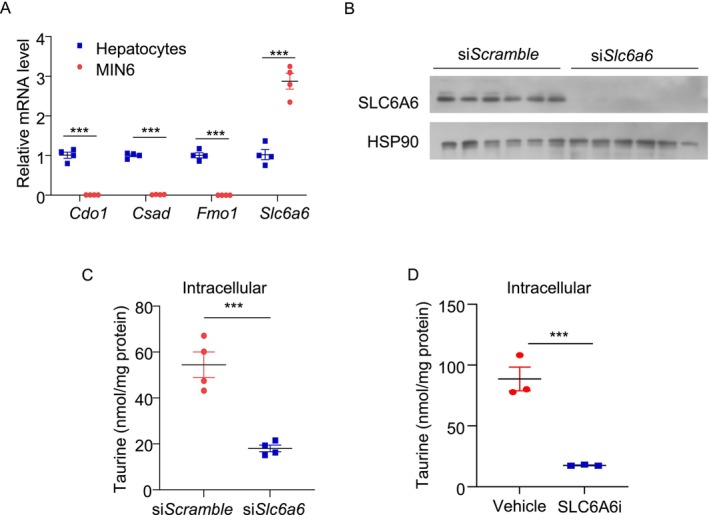
β‐cells acquire taurine through Slc6a6‐mediated uptake. (A) QPCR analysis of taurine biosynthesis related genes and its transporter Slc6a6 in MIN6 cells and mouse hepatocytes. The results are presented as relative levels over respective gene expression in mouse hepatocytes. (*n* = 4). (B, C) MIN6 cells were transfected with siRNA against *Scramble* or *Slc6a6* for 24 h, followed by treatment with taurine (100 μM) or vehicle for 24 h. (B) Immunoblotting analysis of SLC6A6 protein level in each group. (*n* = 3). (C) Intracellular taurine levels in the transfected MIN6 cells. (*n* = 4). (D) MIN6 cells were pre‐treated with non‐FBS culture medium. The cells were then treated with taurine (100 μM) for 24 h, followed by treatment with SLC6A6 inhibitor (SLC6A6i) (100 μM) or vehicle for 30 min. Intracellular taurine concentration was measured by LC–MS/MS. (*n* = 3). All results are presented as mean ± SEM. Significance was determined using two‐tailed independent student's *t*‐test. *p < 0.05, **p < 0.01, ***p < 0.001.

Taurine can be acquired through two pathways: endogenous biosynthesis and exogenous intake. While taurine is predominantly synthesized in the liver, other tissues primarily rely on exogenous uptake [37, 38]. We measured the key genes related to taurine biosynthesis and uptake in MIN6 cells and compared their expression with that in mouse primary hepatocytes. QPCR results indicated that the expression levels of taurine biosynthetic genes (including *Cdo1*, *Csad*, and *Fmo1*) were over 99% lower in MIN6 cells compared to hepatocytes. In contrast, the expression level of the well‐known taurine transporter *Slc6a6* was approximately three times higher in MIN6 cells than in hepatocytes (Figure 5A). siRNA‐mediated silencing of SLC6A6 or treatment with the SLC6A6 inhibitor (guanidinoethyl sulfonate) dramatically reduced intracellular taurine levels in MIN6 cells (Figure 5B–D). Furthermore, incubating MIN6 cells with hypotaurine, a precursor of taurine, was unable to block doxorubicin‐induced senescent responses (Figure S2A,B). These findings suggest that taurine in β‐cells is mainly derived from extracellular sources.

**FIGURE 6:**
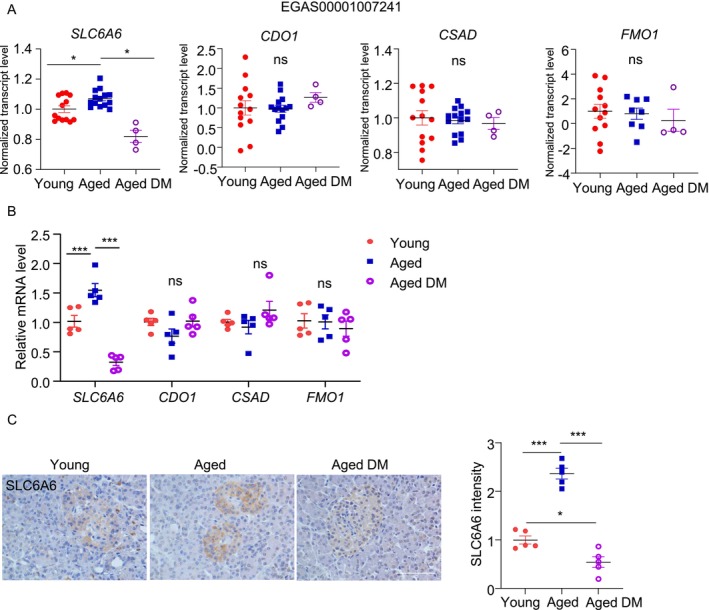
Dynamics of taurine transporter SLC6A6 in pancreatic islets show elevation during natural aging and a significant decrease in diabetic humans. (A) Expression of SLC6A6 and taurine biosynthesis related genes in islets from young (< 35 years, *n* = 13), aged (> 60 years) healthy (*n* = 14) and aged diabetic (DM, *n* = 4) human donors (dataset accession: EGAS00001007241). (B) Pancreatic islets were isolated form young, aged healthy, and aged diabetic male human donors. QPCR analysis of genes related to taurine biosynthesis. (*n* = 5). (C) Representative immunohistochemistry (IHC) staining images of SLC6A6 in pancreatic sections of young, aged healthy, and aged diabetic male human donors, along with the relative SLC6A6 intensity (right panel). (*n* = 5). (Scale bar: 100 μm). All results are presented as mean ± SEM. Significance was determined using one‐way ANOVA with Tukey correction. *p < 0.05, **p < 0.01, ***p < 0.001.

We analyzed publicly available databases to compare gene expressions of the taurine biosynthetic enzymes and transporter in the islets isolated from young (aged < 35 years, *n* = 13) and aged (aged > 60 years, *n* = 14) human subjects with normoglycemia (HbA1c < 5.9%). *SLC6A6* mRNA level was significantly higher in the aged islets compared to the young islets, whereas the taurine biosynthesis genes (*CDO1*, *CSAD*, and *FMO1*) were comparable between the two groups (Figure 6A). Interestingly, when comparing islets from healthy elderly individuals to those with type 2 diabetes (HbA1c ≥ 6.5%), *SLC6A6* was significantly downregulated in diabetic conditions, while the taurine biosynthesis genes remained unchanged (Figure 6A).

To validate these results, we measured these taurine‐related metabolic genes using our in‐house human islet biobank. We only included male subjects and divided them into three groups according to their ages and HbA1c level as follows: 1. Young and non‐DM (24‐38‐year‐old; HbA1c = 5.55 ± 0.40; *n* = 5); 2. Aged and non‐DM (65‐77‐year‐old; HbA1c = 5.50 ± 0.57; *n* = 5) and 3. Aged and DM (63‐70‐year‐old; HbA1c = 8.89 ± 3.06; *n* = 5). QPCR analysis revealed that *SLC6A6* was upregulated in the islets from “Aged and non‐DM” when compared to those from “Young and non‐DM” (Figure 6B). Such aging‐related upregulation was not observed in the aged individuals with DM (Figure 6B). Immunohistochemical staining confirmed the upregulation and downregulation of SLC6A6 protein by aging and diabetic condition, respectively, in humans (Figure 6C). On the contrary, the taurine biosynthetic genes (*CDO1*, *CSAD*, and *FMO1*) were not altered by aging or diabetic condition (Figure 6B). Due to the limited availability of human islets, only the genes and proteins involved in taurine metabolic pathway was assessed; Taurine levels were measured in current study.

**FIGURE 7:**
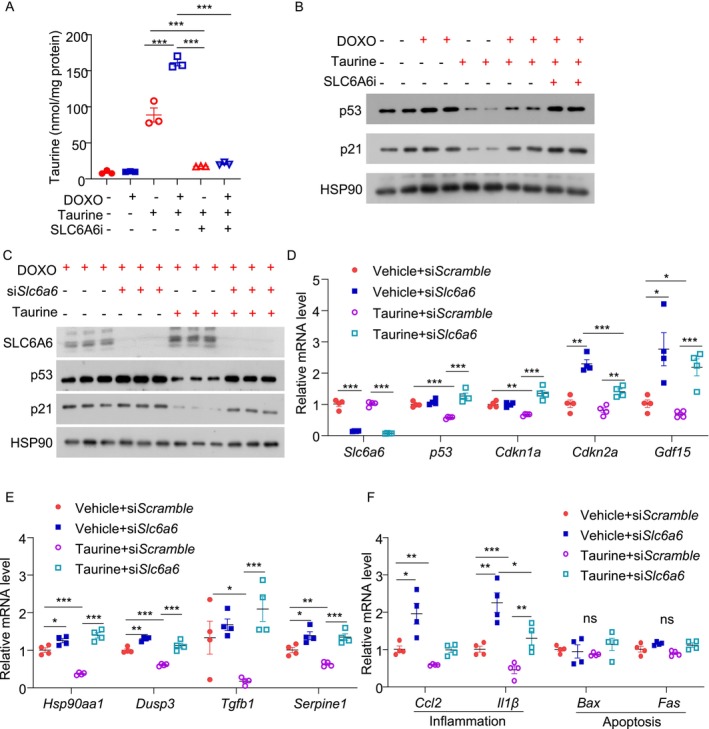
The protective effects of taurine against β‐cell senescence depend on its transporter SLC6A6. (A, B) MIN6 cells were pre‐treated with the SLC6A6 inhibitor (SLC6A6i) (100 μM) or vehicle for 30 min, followed by treatment with taurine (100 μM) and doxorubicin (200 nM) or vehicle for 24 h in non‐FBS culture medium. The intracellular taurine concentration was then measured by LC–MS/MS. (*n* = 3). (B) Immunoblotting analysis of p53 and p21 in each group. (C–F) MIN6 cells were pre‐treated with doxorubicin (200 nM). The cells were then transfected with siRNA against *Scramble* or *Slc6a6* for 24 h, followed by treatment with taurine (100 μM) or vehicle for 24 h. (C) Immunoblotting analysis of SLC6A6, p53, and p21 in each group. (*n* = 3). (D) QPCR analysis of gene expressions related to senescence in each group (*n* = 4). (E) QPCR analysis of the genes related to β‐cell specific SASP in each group. (*n* = 4). (F) QPCR analysis of genes related to inflammation and apoptosis. (*n* = 4). All results are presented as mean ± SEM. Significance was determined using two‐way ANOVA with Tukey correction. *p < 0.05, **p < 0.01, ***p < 0.001.

We next assessed the expression level of SLC6A6 in β‐cells in response to the pro‐aging factors. Both doxorubicin and TNF‐α upregulated SLC6A6 protein levels in MIN6 cells (Figure S3A,B). We hypothesized that the upregulation of SLC6A6 potentially acts as a counteractive response to increase taurine uptake and combat senescence. To test this, we pre‐treated MIN6 cells with the SLC6A6 inhibitor, followed by treatment with taurine and doxorubicin. To eliminate the inference of unknown taurine from serum, we used a serum‐free medium and treated the cells with 100 μM taurine. LC–MS/MS analysis revealed that doxorubicin treatment increased taurine uptake in MIN6 cells, and such increase was completely abolished by the SLC6A6 inhibitor (Figure 7A). Blockage of taurine uptake by the SLC6A6 inhibitor attenuated the inhibitory effects of taurine on doxorubicin‐induced p53 and p21 expression (Figure 7B). Likewise, siRNA‐mediated SLC6A6 silencing diminished the anti‐senescent actions of taurine in response to doxorubicin stimulation in MIN6 cells [21] (Figure 7C–F). These results indicated that taurine primarily relies on exogenous uptake via SLC6A6 to alleviate β‐cell senescence.

### 3.4 The Anti‐Senescent Effects of Taurine Are Mediated by p53 Pathway

**FIGURE 8:**
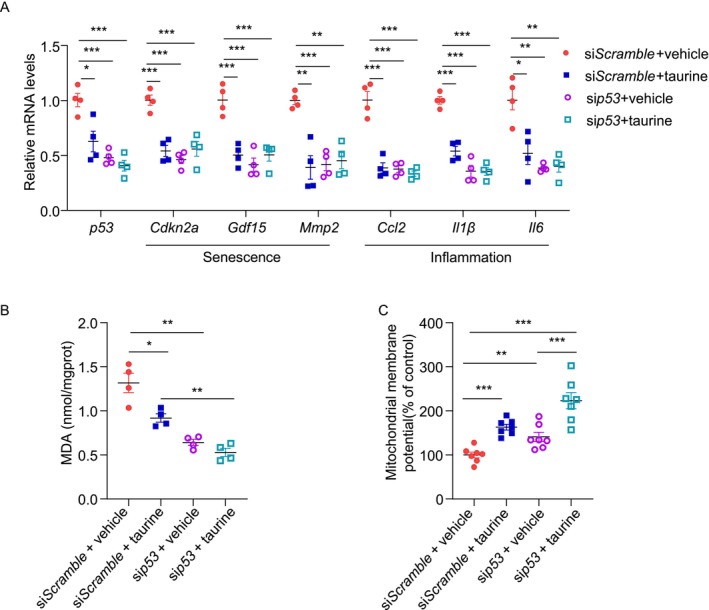
Taurine mitigates senescence, inflammation, and oxidative stress via a p53‐dependent pathway while preserving mitochondrial function independently of p53. (A–C) MIN6 cells were pre‐treated with DOXO (200 nM). The cells were then transfected with siRNA against scramble or p53 for 24 h, followed by treatment with taurine (100 μM) or vehicle for 24 h. Cells were cultured in FBS‐free medium to avoid possible contamination of taurine. (A) QPCR analysis of the genes related to senescence and inflammation in each group. (*n* = 4) Relative mRNA levels were normalized to β‐actin. (B) Cellular content of malondialdehyde (MDA) in each group. (*n* = 4). (C) Mitochondrial membrane potential was measured using TMRE mitochondrial membrane potential assay. (*n* = 7). All results are presented as mean ± SEM. Significance was determined using two‐way ANOVA with Tukey correction. *p < 0.05, **p < 0.005, ***p < 0.001.

p53 activation is known to cause senescence and pancreatic β‐cell dysfunction [13]. To investigate whether the protective effects of taurine on β‐cells are p53‐dependent, we silenced p53 in MIN6 cells using siRNA prior to taurine and doxorubicin treatment. Transfection with siRNA against *p53* successfully downregulated p53 expression in MIN6 cells treated with vehicle, mirroring the effect of taurine treatment. Taurine treatment plus *sip53* transfection did not further reduce p53 expression in MIN6 cells compared to those treated with taurine and transfected with *siScramble*. Taurine treatment reduced the expression of numerous senescent (*Cdkn2a*, *Gdf15*, and *Mmp2*) and inflammatory (*Ccl2*, *Il1β*, and *Il6*) markers in MIN6 cells transfected with *siScramble* (Figure 8A). However, taurine did not reduce the expression of these markers in MIN6 cells transfected with *sip53*, suggesting that its anti‐inflammatory and ‐senescent effects are mainly mediated via p53 (Figure 8A). Taurine is known to reduce oxidative stress in multiple cell types, including pancreatic β‐cells [39, 40]. To evaluate the effect of taurine on oxidative stress, we measured the cellular content of malondialdehyde (MDA), a biomarker of oxidative stress and lipid peroxidation, in MIN6 cells. MIN6 cells were first transfected with *siScramble* or *sip53* for 24 h, followed by taurine and/or doxorubicin treatment for 24 h. Taurine significantly reduced the intracellular MDA level in MIN6 cells transfected with *siScramble*. However, it did not further reduce the MDA level in MIN6 cells with p53 downregulation (Figure 8B). These findings suggest that taurine alleviates oxidative stress via a p53‐dependent manner under doxorubicin‐treated conditions. We also assessed mitochondrial function by measuring mitochondrial membrane potential using tetramethylrhodamine ethyl ester (TMRE) staining. Taurine significantly increased the mitochondrial membrane potential in both *siScramble* and *sip53* transfected MIN6 cells under doxorubicin‐treated conditions. This indicates that the protective effect of taurine on mitochondrial function might be independent of p53 (Figure 8C). Overall, taurine exerts its anti‐senescent, ‐inflammatory, and ‐oxidant effects via a p53‐dependent manner, while its protective effect on mitochondria is p53‐independent.

### 3.5 Taurine Binds to CDKN2AIP, Inhibiting Its Interaction With p53 to Suppress Cellular Senescence

**FIGURE 9:**
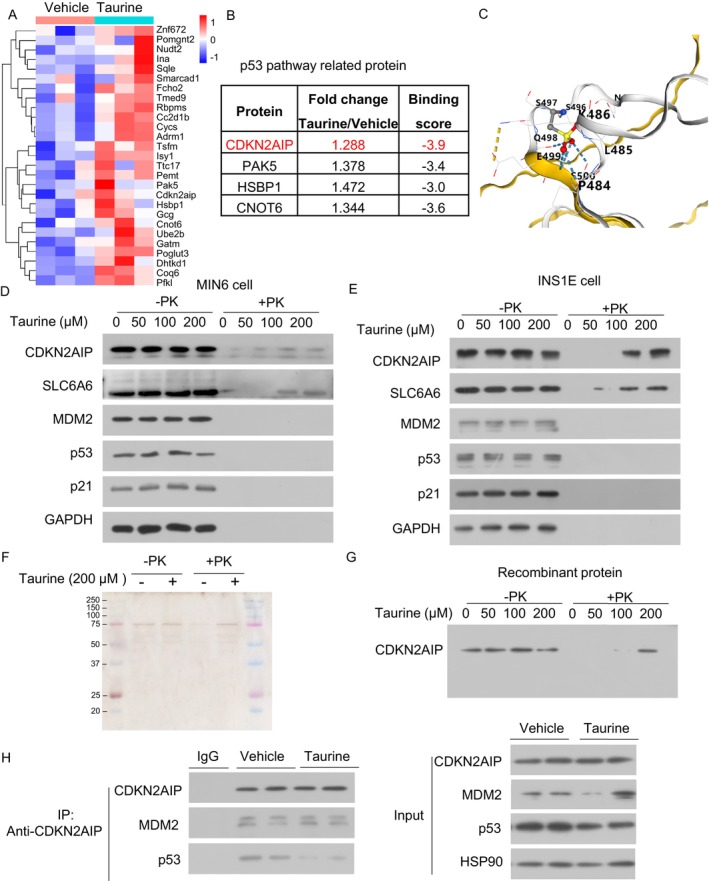
Identification of Taurine‐CDKN2AIP binding in pancreatic β cells. (A, B) Limited proteolysis‐mass spectrometry (LiP‐MS) was used to screen for taurine interacting proteins in the INS1E β‐cell proteome. Heatmap shows potential taurine binding targets identified by LiP‐MS. Vehicle: *N* = 3. Taurine: *N* = 3. (B) p53 pathway related proteins levels between two groups and their binding scores with taurine. (C) Three‐dimensional diagram of the binding modes between human CDKN2AIP and taurine. Taurine potentially binds to CDKN2AIP via residues PRO484, LEU485, LYS486. (D) DARTS analysis using MIN6 cell lysates incubated with taurine. (E) DARTS analysis using INS1E cell lysates incubated with taurine. (F) 500 ng of CDKN2AIP recombinant protein were subjected to SDS‐PAGE and silver staining to assess purity. (G) DARTS analysis using CDKN2AIP recombinant protein incubated with taurine. (H) MIN6 cells treated with taurine (100 μM, 24 h) or vehicle were subjected to immunoprecipitation against CDKN2AIP.

Next, we explored the mechanisms by which taurine inhibits p53 activation and β‐cell senescence. Taurine has been reported to bind to various proteins and modulate their functions [41, 42]. We employed Limited Proteolysis Mass Spectrometry (LiP‐MS) to identify potential taurine‐interacting proteins in β‐cells [43]. Mass spectrometry analysis revealed 35 candidate proteins that may bind to taurine (fold change > 1.2, *p* < 0.05) (Figure 9A). Among these, four proteins were involved in regulating the p53 pathway, which include CDKN2AIP, PAK5, HSBP1, and CNOT6 (Figure 9B). Molecular docking simulations indicated that CDKN2AIP has the highest likelihood of taurine binding, based on calculated binding scores (a more negative value indicate a stronger binding) (Figure 9B). CDKN2AIP, also known as CARF (Collaborator of ARF), is a positive regulator of p53 via binding to p53 and enhancing its stability in response to DNA damage [44, 45, 46]. Using the CB‐DOCK2 molecular docking software, we predicted the binding sites of taurine on CDKN2AIP, primarily at amino acid residues PRO484, LEU485, and LYS486 (Figure 9C). The DARTS assay [23] confirmed that taurine dose‐dependently prevented protease K‐induced degradation of CDKN2AIP and SLC6A6 (as a positive control of taurine binding protein) in lysates from MIN6 and INS‐1E cells. On the contrary, taurine did not interfere with the proteolytic degradation of p53, MDM2 (an E3 ligase targeting p53 degradation) or p21, indicating that they are not the taurine binding partners (Figure 9D,E). To confirm whether the binding of taurine to CDKN2AIP is direct, we incubated recombinant human CDKN2AIP protein with proteinase in the presence or absence of taurine. Taurine also prevented proteinase K‐induced degradation of the CDKN2AIP recombinant protein (Figure 9F,G). CDKN2AIP binds with p53 and increases its stability and expression. Taurine reduced the interaction between p53 and CDKN2AIP in MIN6 cells, and such an effect was accompanied by p53 reduction (Figure 9H). On the other hand, taurine treatment did not modulate p53‐MDM2 expression. These findings demonstrate that taurine suppresses the p53‐mediated senescent response by interrupting the p53‐CDKN2AIP interaction and hence subsequent p53 reduction.

**FIGURE 10:**
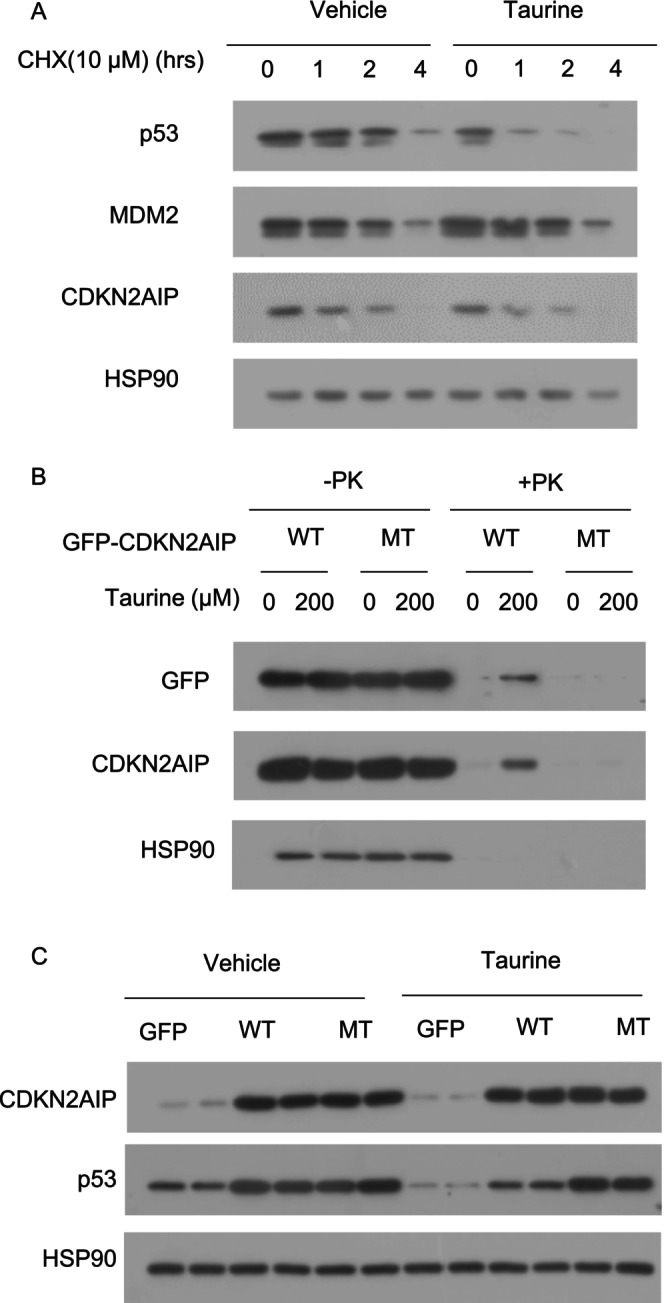
Taurine treatment accelerates p53 degradation by binding to CDKN2AIP. (A) p53 protein degradation was detected using cycloheximide (CHX, 10 μM) chase assay. (B) HEK 293 cells were transfected with plasmids encoding GFP‐tagged CDKN2AIP (WT) and CDKN2AIP‐triple mutant (MT) for 48 h. DARTS analysis was performed using cell lysates incubated with taurine, followed by immunoblotting analysis as indicated. (C) INS‐1E cells were transfected with plasmids encoding GFP control, GFP‐tagged CDKN2AIP, and its triple mutant for 24 h, followed by taurine treatment for 24 h. Immunoblotting analysis of CDKN2AIP and p53 in each group.

To further confirm the effect of taurine on p53 protein stability, we conducted a cycloheximide (CHX) chase assay. The results showed that taurine treatment significantly accelerated p53 protein degradation without affecting the stability of CDKN2AIP or MDM2 (Figure 10A). To validate the binding sites, we generated a plasmid expressing a CDKN2AIP mutant in which PRO484, LEU485, and LYS486 were mutated to alanine residues (CDKN2AIP‐triple mutant). DARTS experiments demonstrated that taurine bound to wild‐type CDKN2AIP but not the CDKN2AIP‐triple mutant (Figure 10B), suggesting taurine binds to CDKN2AIP through PRO484, LEU485, and LYS486.

Next, we determined whether the binding of taurine to CDKN2AIP is necessary for the inhibitory effect of taurine on p53 expression. Overexpression of wild‐type CDKN2AIP upregulated p53 protein expression, but this upregulation was counteracted by taurine treatment. In contrast, taurine was unable to counteract p53 upregulation induced by the CDKN2AIP‐triple mutant (Figure 10C). These findings indicate that the interaction between taurine and CDKN2AIP is essential for restricting p53 expression in β‐cells.

## 4 Discussion

The accumulation of senescent pancreatic β‐cells contributes to age‐related diabetes mellitus. These senescent cells are characterized by loss of β‐cell identity, cell cycle arrest, and the secretion of pro‐inflammatory SASP factors, which collectively compromise β‐cell function and viability. In this study, we identified increased taurine uptake in aging β‐cells as a protective mechanism against cellular senescence through suppression of the p53 pathway. Reduced expression of the transporter SLC6A6 impairs taurine uptake, promoting senescence and β‐cell dysfunction.

The beneficial effects of taurine on pancreatic β‐cell function are well‐documented. Taurine has been shown to modulate intracellular calcium levels, facilitating insulin secretion [47, 48]. Furthermore, taurine inhibits the release of pro‐inflammatory cytokines, thereby alleviating pancreatic inflammation. Studies have shown that taurine reduces levels of TNF‐α and IL‐6, both of which play key roles in the pathology of diabetes and cellular senescence [48, 49]. Additionally, taurine promotes β‐cell survival and proliferation while mitigating apoptosis by activating various signaling pathways [40, 48, 50, 51, 52, 53, 54]. Beyond pancreatic β‐cells, taurine has been observed to inhibit cellular senescence in other tissues, such as muscle and liver, by modulating oxidative stress and inflammatory responses [28, 29, 30]. This suggests that taurine's protective effects against senescence may be a broader phenomenon, potentially applicable to various cell types and tissues. To our best knowledge, the anti‐senescent effect of taurine on β‐cells has not been reported previously. In the context of aging, distinguishing between healthy and unhealthy aging is essential for understanding the pathophysiology of diabetes. Healthy aging is characterized by sustained physiological function, resilience to stress, and effective tissue repair mechanisms that collectively support the preservation of β‐cell function. Our research indicates that taurine plays a significant role in enhancing compensatory uptake in β‐cells during healthy aging, facilitating the maintenance of normal physiological function.

Taurine metabolism in the human body primarily takes place in the liver, where it is synthesized from cysteine and other precursors. Taurine then circulates and is uptaken by various tissues, including muscle, adipose tissue, and the pancreatic islet, via the taurine transporter SLC6A6 [27, 55, 56]. The intracellular level of taurine is tightly regulated by the expression of its transporter SLC6A6 in non‐hepatocytes. However, as individuals age, systemic taurine levels tend to decline due to several factors, including reduced endogenous synthesis and impaired transport capacity [28]. Specifically, the expression of SLC6A6, which facilitates taurine uptake, has been shown to be downregulated under certain conditions, such as oxidative stress or chronic inflammation, which are common in aging and metabolic diseases [57, 58]. Additionally, the absence of 3‐phosphoinositide‐dependent protein kinase‐1 (Pdk1), a known regulator of cell proliferation and death, was identified to disrupt taurine homeostasis via reducing SLC6A6 protein expression in the heart, underscoring its significance in maintaining cardiomyocyte survival [59]. Conversely, overexpression of SLC6A6 reduces reactive oxygen species (ROS) production [60]. Our study points out that the endogenous synthesis of taurine in β‐cells is extremely limited, with these cells mainly relying on the transporter SLC6A6 for exogenous uptake. This finding fills a key gap in the understanding of taurine metabolism in pancreatic β‐cells. During naturally aging, the upregulation of SLC6A6‐mediated taurine uptake in β‐cells acts as a compensatory response to decreased circulating taurine levels. However, this upregulation is absent in islets from aged individuals with diabetes, suggesting that an inability to maintain this transporter‐mediated taurine uptake may contribute to diabetes development in the elderly. Not only is taurine supplementation important, but the restoration of transporter expression is also crucial.

p53 protein plays a pivotal role in regulating pancreatic β‐cell function, particularly in the context of cellular senescence [12, 13, 61]. As a critical tumor suppressor, p53 is involved in various cellular processes, including cell cycle regulation, apoptosis, and DNA repair [13]. In β‐cells, p53 activation in response to stressors such as oxidative stress or DNA damage can lead to cellular senescence, which is characterized by a permanent cell cycle arrest and altered secretory function. In our study, we identified several novel taurine binding proteins that are intricately related to the p53 signaling pathway, including CDKN2AIP, PAK5, HSBP1, and CNOT6. One of the key proteins we focused on is CDKN2AIP. As a collaborating factor of ARF, CDKN2AIP plays a crucial role in regulating the stability and activity of p53, thereby affecting cell proliferation, apoptosis, and senescence processes [44, 45, 62]. This regulatory mechanism is particularly important when cells respond to DNA damage and other stresses because the activation of p53 can initiate cell repair mechanisms and prevent potential tumorigenesis. Interestingly, we found that this p53‐CDKN2AIP protein interaction is regulated by taurine in β‐cells. We performed a LiP‐MS assay to identify the potential binding relationship between taurine and CDKN2AIP, predicted their binding potential using molecular docking simulation, and conclusively verified their interaction through DARTS analysis and CO‐IP experiments. In‐depth research found that the binding of taurine to CDKN2AIP inhibits its interaction with p53, ultimately destabilizing the p53 protein and decreasing the expression of senescence‐ and inflammatory response‐related markers. Similarly, taurine was reported to interact with various proteins, influencing their conformation and activity. For example, taurine enhances the binding of both mutant and wild‐type p53 to DNA, suggesting that taurine may alter p53 protein conformation to facilitate its interaction with DNA [63]. Taurine was also reported to induce an ordered but functionally inactive conformation in casein protein [64]. Furthermore, taurine interacts with liver X receptor α (LXRα) during lipid metabolism to act as a ligand [65]. Importantly, our research identified CDKN2AIP as a novel taurine targeting protein, highlighting another layer of interaction that may influence p53 activity. Our study elucidates a previously unrecognized regulatory axis wherein taurine directly binds CDKN2AIP through three critical residues (Pro^484^, Leu^485^, and Lys^486^) to modulate p53 protein dynamics in β‐cells. These findings collectively support the diverse protein‐binding capabilities and biological functions of taurine.

Despite the significant findings of our study, several limitations should be acknowledged. Notably, interventions targeting the clearance of senescent cells have been shown to effectively improve β‐cell function and glucose metabolism, underscoring the critical role of β‐cell senescence in diabetes pathophysiology [35, 66]. However, we didn't have in vivo animal experiments to assess the effects of taurine supplementation in a living organism, which limits the translation of our results to potential therapeutic applications. Therefore, our research holds potential therapeutic value that warrants further exploration. Our study demonstrates taurine's anti‐senescence effects in vitro and in murine islets. However, the anti‐senescence and ‐diabetic effects of taurine need to be further validated using diabetic animal models under aging conditions. Additionally, the mechanism by which SLC6A6 expression changes differently in β‐cells during aging and diabetes remains unclear. Further investigation is needed, as understanding this process is crucial for optimizing the beneficial effects of taurine supplementation. Finally, our study lacks direct testing of taurine's effects on human β‐cells and human islets, which is vital for understanding its therapeutic potential in human metabolic diseases, limiting the translation of our findings to clinical applications, and necessitating further research in human models.

In summary, this study demonstrates that taurine protects against the senescence of pancreatic β‐cells through SLC6A6‐mediated uptake. Exogenous taurine supplementation reduces the expression of senescence‐related markers. By binding to CDKN2AIP, taurine regulates p53 activity, destabilizes the p53 protein, thereby inhibiting β‐cell senescence and delaying the onset of diabetes.

## Author Contributions

Baomin Wang performed most of the experiments, drafted, and revised the manuscript. Ziyan Wang and Yumei Yang performed several important parts of the experiments and helped to revise the manuscript. Melody Yuen Man Ho carried out the metabolomics experiments. Runyue Yang and Huizi Yang helped to analyze the bioinformatic data. Siyi Liu collected human pancreas tissues. Huige Lin, Kenneth King Yip Cheng, and Xiaomu Li advised the study design, supervised the study, provided important resources, acquired funding, revised, and finalized the manuscript.

## Disclosure

The authors have nothing to report.

## Conflicts of Interest

The authors declare no conflicts of interest.

## Supporting information


**Data S1:** Supporting Information.

## Data Availability

Data will be available upon reasonable request.
