# Supplementary material for: Taurine Alleviates Pancreatic β‐Cell Senescence by Inhibition of p53 Pathway
Source: J Diabetes. 2025 Jun 3;17(6):e70100. doi: 10.1111/1753-0407.70100 (PMC12130737; doi:10.1111/1753-0407.70100)
Supplement: Supplementary file 1 — Data S1: Supporting Information. [file JDB-17-e70100-s001.pdf]

Supplementary Figure 1

A

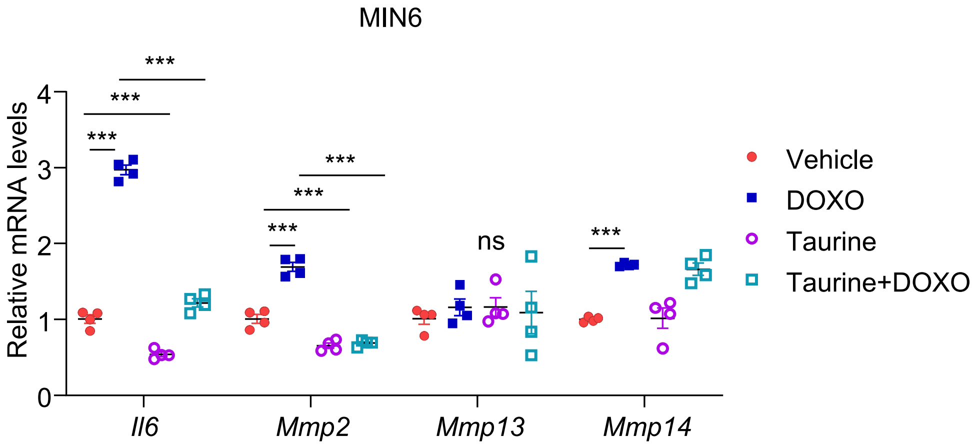

B

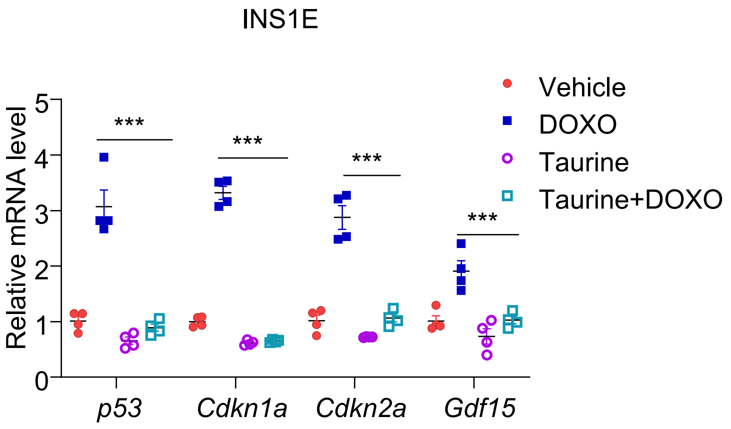

Figure S1. Taurine supplementation alleviates doxorubicin-induced MIN6 and INS1E β-cell senescence.
(A) MIN6 cells were pre-treated with 100 µM taurine for 24 hours, followed by 200 nM doxorubicin (DOXO) treatment for 24 hours. Cells were cultured in FBS-free medium to avoid possible contamination of taurine. QPCR analysis of the genes *Il6*, *Mmp2*, *Mmp13* and *Mmp14* in each group of doxorubicin-induced senescence model. (n=4) Relative mRNA levels were normalized to β-actin. (B) INS1E cells were pre-treated with 100 µM taurine for 24 hours, followed by 200 nM doxorubicin (DOXO) treatment for 24 hours. Cells were cultured in FBS-free medium to avoid possible contamination of taurine. QPCR analysis of the genes related to senescence in each group. (n=4). All results are presented as mean ± SEM. Significance was determined using two-way ANOVA with Tukey correction. * $p<0.05$, ** $p<0.01$, *** $p<0.001$.

Supplementary Figure 2

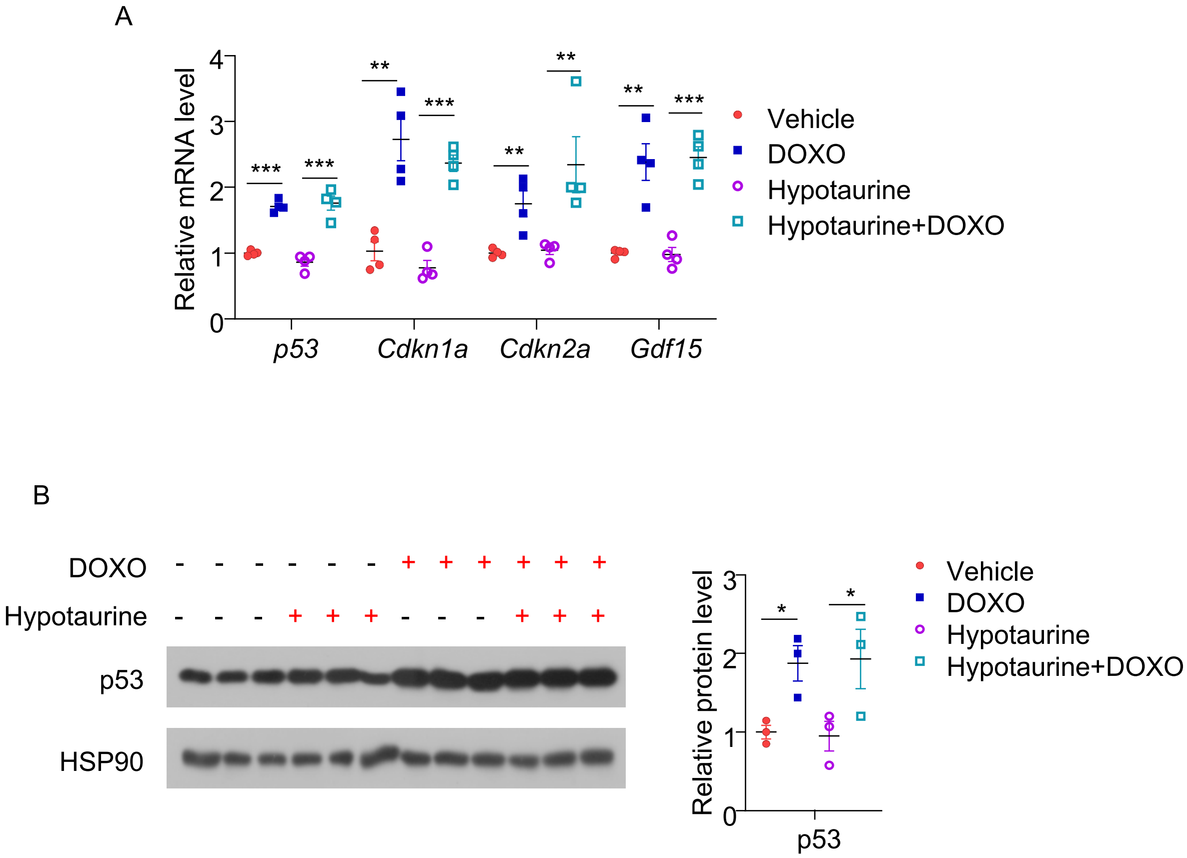

Figure S2. Hypotaurine supplementation cannot alleviate doxorubicin-induced β-cell senescence.
MIN6 cells were pre-treated with 100 μM hypotaurine for 24 hours, followed by 200 nM doxorubicin treatment for 24 hours. Cells were cultured in FBS-free medium to avoid possible contamination of hypotaurine. (A) QPCR analysis of the genes related to senescence in each group in doxorubicin-induced senescence model. (n=4). (B) Immunoblotting analysis of p53 and p21. (n=3). All results are presented as mean ± SEM. Significance was determined using two-way ANOVA with Tukey correction. * $p<0.05$, ** $p<0.01$, *** $p<0.001$.

A

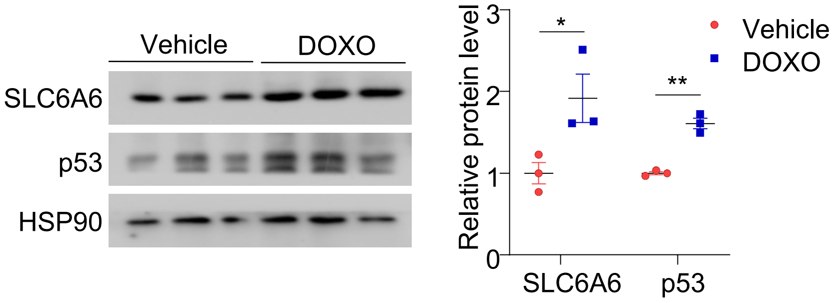

B

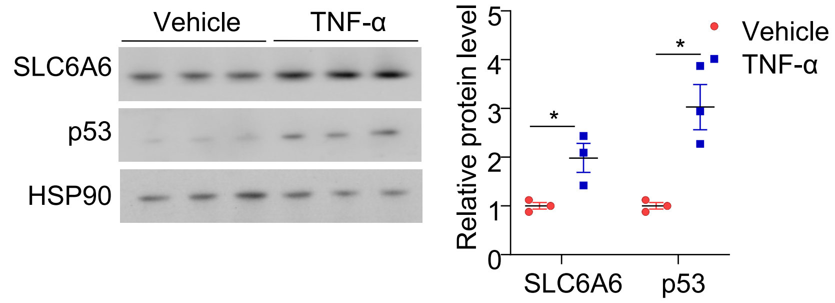

Figure S3. Doxorubicin and TNF-α upregulate SLC6A6 expression in MIN6 cells.
(A) Immunoblotting analysis of SLC6A6 protein level in doxorubicin (200 nM, 24 hours)-induced MIN6 cells senescence condition. (n=3). (B) Immunoblotting analysis of SLC6A6 level in TNF-α (20 ng/ml, 24 hours)-induced MIN6 cells senescence conditions. (n=3). All results are presented as mean ± SEM. Significance was determined using two-tailed independent student's t-test. * $p<0.05$, ** $p<0.005$, *** $p<0.001$.

Supplementary Table 1. Information of Human Pancreas Donors in Figure 6B-D

Human donors related to this paper

Donor	Gender	Age (yrs)	Height (cm)	Weight (kg)	BMI (kg/m²)	HbA1c (%)	Group	Cause of death
1(Fig.6B-D)	M	34	175	65	21.22	5.88	Young & non-DM	Head Trauma
2(Fig.6B-D)	M	35	170	70	24.22	5.02	Young & non-DM	Head Trauma
3(Fig.6B-D)	M	24	170	65	22.49	5.32	Young & non-DM	Stroke
4(Fig.6B-D)	M	34	175	61	19.92	5.53	Young & non-DM	Head Trauma
5(Fig.6B-D)	M	28	165	55	20.20	6.00	Young & non-DM	Stroke
6(Fig.6B-D)	M	69	180	85	26.23	4.79	Aged & non-DM	Head Trauma
7(Fig.6B-D)	M	71	165	65	25.39	4.99	Aged & non-DM	Head Trauma
8(Fig.6B-D)	M	77	170	70	24.22	5.89	Aged & non-DM	Head Trauma
9(Fig.6B-D)	M	68	170	70	24.22	6.09	Aged & non-DM	Stroke
10(Fig.6B-D)	M	65	175	75	24.49	5.72	Aged & non-DM	Stroke
11(Fig.6B-D)	M	67	158	54	21.63	13.20	Aged & DM	Head Trauma
12(Fig.6B-D)	M	64	175	75	24.49	11.1	Aged & DM	Stroke
13(Fig.6B-D)	M	67	170	65	22.49	6.60	Aged & DM	Head Trauma
14(Fig.6B-D)	M	70	170	70	24.22	6.80	Aged & DM	Stroke
15(Fig.6B-D)	M	63	175	70	22.86	6.77	Aged & DM	Head Trauma

Supplementary Table 2. Characteristics of the study participants in Figure 6B-D.

Group	Age (yrs) (mean ± SEM)	HbA1c (%) (mean ± SEM)	BMI (kg/m²) (mean ± SEM)
Young & non-DM	31.00 ± 4.80	5.55 ± 0.40	21.61 ± 1.77
Aged & non-DM	70.00 ± 4.47	5.50 ± 0.57	24.91 ± 0.88
Aged & DM	66.20 ± 2.77	8.89 ± 3.06	23.14 ± 1.20
p-value (Young & non-DM vs Aged & non-DM)	<0.001***	0.87	0.01*
p-value (Young & non-DM vs Aged & DM)	<0.001***	0.04*	0.15
p-value (Aged & non-DM vs Aged & DM)	0.15	0.04*	0.03*

Supplementary Table 3. Sequences of siRNA

Gene	Sense(5'-3')	Antisense(5'-3')
siRNA against mouse *Scl6a6*	GGUCCAGCAAGAUCGACUUTT	GUUGUCUACUUCACCGCUATT

Supplementary Table 4. Sequences of Primers

**QPCR**		
p53 (m)	ACTGCATGGACGATCTGTTG	GTGACAGGGTCCTGTGCTG
Cdkn1α (m)	TAGCCTGAATACTCGCCTCTT	GCCAGGGCTTTGATGTGC
Cdkn2a(m)	CGCAGGTTCTTGGTCACTGT	TGTTCACGAAAGCCAGAGCG
Gdf15(m)	CTGGCAATGCCTGAACAACG	GGTCGGGACTTGGTTCTGAG
Ccl2 (m)	CACTCACCTGCTGCTACTCA	GCTTGGTGACAAAAACTACAGC
Il1β (m)	GCAACTGTTCCTGAACTCAACT	ATCTTTTGGGGTCCGTCAACT
Bax (m)	TGAAGACAGGGGCCTTTTTG	AATTCGCCGGAGACACTCG
Fas (m)	TATCAAGGAGGCCCATTTTGC	TGTTTCCACTTCTAAACCATGCT
Cdo1 (m)	GGGGACGAAGTCAACGTGG	ACCCCAGCACAGAATCATCAG
Csad (m)	CCAGGACGTGTTTGGGATTGT	ACCAGTCTTGACACTGTAGTGA
Fmo1 (m)	ACAGCCGACAGTATAAACATCCA	CCCTCCAGTAGTGCTGAGGAA
Slc6a6 (m)	GCACACGGCCTGAAGATGA	ATTTTTGTAGCAGAGGTACGGG
Hsp90aa1 (m)	TGTTGCGGTACTACACATCTGC	GTCCTTGGTCTCACCTGTGATA
Dusp3 (m)	TCTGTGGCTCAGGACATCAC	GGCCCTTTCAAAGTAAGCACTG
Tgfb1 (m)	CTCCCGTGGCTTCTAGTGC	GCCTTAGTTTGGACAGGATCTG
Serpine1 (m)	TTCAGCCCTTGCTTGCCTC	ACACTTTTACTCCGAAGTCGGT
Actin (m)	CGCCACCAGTTCGCCATGGA	TACAGCCCGGGGAGCATCGT
36b4 (m)	AGATTCGGGATATGCTGTTGGC	TCGGGTCCTAGACCAGTGTTC
CDO1 (h)	ATGGAACAGACCGAAGTGCTG	CTTGGCGTACATTGCCCAC
CSAD (h)	CTTCTCCAGGATACCTCGAACC	CAGAGCCACATCGTAGAACTTG
FMO1 (h)	GCCAAGCGAGTTGCCATTG	CACAGACTTGTAGAGACTGGCT
SLC6A6 (h)	GGAGAAGTGGTCTAGCAAGATCG	AGAAACGCACCTCCACCATTC
18S (h)	GTAACCCGTTGAACCCCATT	CCATCCAATCGGTAGTAGCG
TP53 (h)	CAGCACATGACGGAGGTTGT	TCATCCAAATACTCCACACGC
CDKN1A (h)	TGTCCGTCAGAACCCATGC	AAAGTCGAAGTTCCATCGCTC
ACTIN (h)	GGCATTCACGAGACCACCTAC	CGACATGACGTTGTTGGCATAC
